# Limitations of the incidence density ratio as approximation of the hazard ratio

**DOI:** 10.1186/s13063-019-3590-2

**Published:** 2019-08-08

**Authors:** Ralf Bender, Lars Beckmann

**Affiliations:** 1 Department of Medical Biometry, Institute for Quality and Efficiency in Health Care (IQWiG), Im Mediapark 8, D–50670 Cologne, Germany; 2 Faculty of Medicine, University of Cologne, Cologne, Germany

**Keywords:** Hazard function, Incidence rate, Incidence density ratio, Randomized controlled trials, Simulation, Time-to-event data

## Abstract

**Background:**

Incidence density ratios (IDRs) are frequently used to account for varying follow-up times when comparing the risks of adverse events in two treatment groups. The validity of the IDR as approximation of the hazard ratio (HR) is unknown in the situation of differential average follow up by treatment group and non-constant hazard functions. Thus, the use of the IDR when individual patient data are not available might be questionable.

**Methods:**

A simulation study was performed using various survival-time distributions with increasing and decreasing hazard functions and various situations of differential follow up by treatment group. HRs and IDRs were estimated from the simulated survival times and compared with the true HR. A rule of thumb was derived to decide in which data situations the IDR can be used as approximation of the HR.

**Results:**

The results show that the validity of the IDR depends on the survival-time distribution, the difference between the average follow-up durations, the baseline risk, and the sample size. For non-constant hazard functions, the IDR is only an adequate approximation of the HR if the average follow-up durations of the groups are equal and the baseline risk is not larger than 25%. In the case of large differences in the average follow-up durations between the groups and non-constant hazard functions, the IDR represents no valid approximation of the HR.

**Conclusions:**

The proposed rule of thumb allows the use of the IDR as approximation of the HR in specific data situations, when it is not possible to estimate the HR by means of adequate survival-time methods because the required individual patient data are not available. However, in general, adequate survival-time methods should be used to analyze adverse events rather than the simple IDR.

## Background

Adverse events play an important role in the assessment of medical interventions. Simple standard methods for contingency tables are frequently applied for the analysis of adverse events. However, the application of simple, standard methods may be misleading if observations are censored at the time of discontinuation due to, for example, treatment switching or noncompliance, resulting in varying follow-up times, which sometimes differ remarkably between treatment groups [1]. Incidence densities (IDs), i.e., events per patient years, are frequently used to account for varying follow-up times when quantifying the risk of adverse events [2–4]. IDs are also called exposure-adjusted incidence rates (EAIRs) to underline that varying follow-up times are taken into account [2–5]. For comparisons between groups, incidence density ratios (IDRs) are used together with confidence intervals (CIs) based upon the assumption that the corresponding time-to-event variables follow an exponential distribution. The corresponding results are interpreted in the same way as hazard ratios (HRs).

An example is given by the benefit assessment of the Institute for Quality and Efficiency in Health Care (IQWiG) in which the added benefit of abiraterone acetate (abiraterone for short) in comparison with watchful waiting was investigated in men with metastatic prostate cancer that is not susceptible to hormone-blocking therapy, who have no symptoms or only mild ones, and in whom chemotherapy is not yet indicated [6]. In this report the IDR was used to compare the risks of cardiac failure in the abiraterone group and the control group of the corresponding approval study. The result was IDR = 4.20, 95% CI 0.94, 18.76; *P* = 0.060. It is questionable whether the use of the IDR is adequate in this data situation because the median follow-up duration was 14.8 months in the abiraterone group but only 9.3 months in the control group. The reason for this large difference was the discontinuation of treatment after disease progression with stopping of the monitoring of adverse events 30 days later. In the situation of constant hazard functions, i.e., if the time-to-event data follow an exponential distribution, the IDR accounts for the differential follow up by treatment group. However, if the hazard functions are not constant, the effect of differential follow up by treatment group on the behavior of the IDR is unknown. Appropriate methods should be used for analysis of survival data if access to the individual patient data is available. However, access to the individual patient data is not available in the assessment of dossiers or publications with aggregate-level data. In this situation, a decision has to be made on the situations in which the IDR can or cannot be used as adequate approximation for the HR.

The use of IDs makes sense in the situation of constant hazard functions in both groups [2, 3, 5, 7]. However, time-to-event data rarely follow an exponential distribution in medical research [3, 7]. In the case of low event risks, deviations from the exponential distribution may be negligible if the average follow up is comparable in both groups [2]. However, in the case of differential follow up by treatment group, deviations from the exponential distribution may have a considerable effect on the validity of the IDR and the corresponding CIs as an approximation of the HR.

Kunz et al. [8] investigated bias and coverage probability (CP) of point and interval estimates of IDR in meta-analyses and in a single study with differential follow up by treatment group when incorrectly assuming that average follow up is equal in the two groups. It was shown that bias and CP worsen rapidly with increasing difference in the average follow-up durations between the groups [8]. Here, we do not consider the effect of incorrectly assuming equal average follow-up durations. IDR is calculated correctly by using the different follow-up durations in the groups. The focus here is the effect of deviations from the exponential distribution of the time-to-event data.

In this paper, the validity of the IDR as approximation of the HR is investigated in the situation of differential average follow up by treatment group by means of a simulation study considering decreasing and increasing hazard functions. A rule of thumb is derived to decide in which data situations the IDR can be used as approximation of the HR. We illustrate the application of the rule by using a real data example.

## Methods

### Data generation

We considered the situation of a randomized controlled trial (RCT) with two parallel groups of equal sample size *n* in each group. We generated data for a time-to-event variable *T* (time to an absorbing event or time to first event) with a non-constant hazard function according to Bender et al. [9]. The Weibull distribution is used to generate data with decreasing and the Gompertz distribution is used to generate data with increasing hazard functions. The survival functions *S*_*0*_(t)_*weib*_ and *S*_*0*_(t)_*gomp*_ of the control group using the Weibull and the Gompertz distribution, respectively, are defined by:

1 $$ {S}_0{\left(\mathrm{t}\right)}_{weib}=\mathit{\exp}\left(-\uplambda {\mathrm{t}}^{\nu}\right) $$

2 $$ {S}_0{\left(\mathrm{t}\right)}_{gomp}=\mathit{\exp}\left(\frac{\uplambda}{\upalpha}\left(1-\mathit{\exp}\Big(\upalpha \mathrm{t}\right)\right), $$

where λ > 0 is the scale parameter and *ν* > 0, α ∈ (−∞,∞) are the shape parameters of the survival time distributions. The corresponding hazard functions of the control group are given by:

3 $$ {h}_0{\left(\mathrm{t}\right)}_{weib}=\lambda \kern0.5em v\kern0.5em {\mathrm{t}}^{\mathrm{v}-1} $$

4 $$ {h}_0{\left(\mathrm{t}\right)}_{gomp}=\lambda \kern0.5em \mathit{\exp}\left(\upalpha \mathrm{t}\right), $$

leading to a decreasing hazard function for ν < 1 (Weibull), and an increasing hazard function for α > 0 (Gompertz).

We simulated data situations with identical and with different average follow-up durations in the control and intervention group. The average follow-up duration in the control group relative to the intervention group varied from 100% to 30% (in steps of 10%, i.e., 8 scenarios). To simulate a variety of study situations, we chose 9 different baseline risks (BLRs) (BLR = 0.01, 0.02, 0.05, 0.075, 0.1, 0.15, 0.2, 0.25, and 0.3), 7 different effect sizes (HR = 0.4, 0.7, 0.9, 1, 1.11, 1.43, and 2.5), and 3 different sample sizes (*N *= 200, 500, and 1000, with 1:1 randomization). The BLR is the absolute risk of an event in the control group over the actual follow-up period in the control group. The parameters of the survival-time distributions were chosen so that the specified baseline risks and effect sizes are valid for the corresponding follow-up duration in the control group and the HR for the comparison treatment versus control, respectively. We considered 1 situation with decreasing hazard function (Weibull distribution with shape parameter *ν* = 0.75) and 3 different situations with increasing hazard function (Gompertz distribution with shape parameter α = 0.5, 0.75, 1) because the case of increasing hazard was expected to be the more problematic one. The corresponding scale parameters λ for both the Weibull and the Gompertz distribution varied depending on the baseline risk and the follow-up duration in the control group.

First results showed that in some situations with relative average follow-up durations in the control group of 80%, 90%, and 100%, the IDR has adequate properties for all baseline risks considered. Therefore, additional simulations were performed in these cases with larger baseline risks (0.5, 0.7, 0.9, 0.95, and 0.99). In total, the combination of 4 survival distributions with 8 or 3 relative follow-up durations, 9 or 5 baseline risks, 7 effect sizes, and 3 sample sizes resulted in (4 × 8 × 9 × 7 × 3) + (4 × 3 × 5 × 7 × 3) = 7308 different data situations.

We included only simulation runs in which at least 1 event occurred in both groups and the estimation algorithm of the Cox proportional hazard model converged. If at least one of these conditions was violated a new simulation run was started, so that for each of the 7308 data situations 1000 simulation runs were available. This procedure leads to a bias in situations in which simulation runs frequently had to be repeated (very low baseline risk, low sample size). However, this problem concerns both IDR and HR and it was not the goal of the study to evaluate the absolute bias of the estimators.

### Data analysis

The IDR was calculated from the simulated time-to-event data by:

5 $$ \mathrm{IDR}=\frac{{\mathrm{e}}_1/{\sum}_{\mathrm{j}=1}^{\mathrm{n}}{\mathrm{t}}_{1\mathrm{j}}}{{\mathrm{e}}_0/{\sum}_{\mathrm{j}=1}^{\mathrm{n}}{\mathrm{t}}_{0\mathrm{j}}}=\frac{{\mathrm{e}}_1{\sum}_{\mathrm{j}=1}^{\mathrm{n}}{\mathrm{t}}_{0\mathrm{j}}}{{\mathrm{e}}_0{\sum}_{\mathrm{j}=1}^{\mathrm{n}}{\mathrm{t}}_{1\mathrm{j}}}, $$

where e_i_ represents the number of events in the control (i = 0) and the intervention group (i = 1), respectively, and t_ij_ represents the time to event or to study ending in patient j (j = 1, …,*n*) in group i (i = 0,1).

A 95% CI for IDR based on the assumption of a constant hazard function was obtained according to Deeks et al. [10] by:

6 $$ \mathrm{IDR}\pm \exp \Big({\mathrm{z}}_{0.975}\times \mathrm{SE}\left(\log \left(\mathrm{IDR}\right)\right), $$

where z_0.975_ = Φ^−1^(0.975) and Φ denotes the cumulative density function of the standard normal distribution. The standard error (SE) of *log* (IDR) is given by:

7 $$ \mathrm{SE}\left(\log \left(\mathrm{IDR}\right)\right)=\sqrt{\frac{1}{e_1}+\frac{1}{e_0}}. $$

The Cox proportional hazards model was used for point and interval estimation of the HR. All analyses were performed using the R statistical package [11].

### Performance measures

To assess the adequacy of the IDR as approximation of the HR in the situation of non-constant hazard functions we calculated the coverage probability (CP) of the 95% CIs and the mean square error (MSE) and the SE of the point estimates *log* (IDR) and *log* (HR). For effect sizes not equal to 1 (i.e., true HR ≠ 1), additionally the relative bias was calculated. The relative bias is given by the mean percent error (MPE) defined by:

8 $$ \mathrm{MPE}=100\frac{1}{\mathrm{s}}\sum \limits_{\mathrm{j}=1}^{\mathrm{s}}\frac{\theta_{\mathrm{j}}-{\theta}_{\mathrm{true}}}{\theta_{\mathrm{true}}}, $$

where s is the number of simulation runs (s = 1000), θ_j_ is the estimate of the considered parameter in simulation j, and θ_true_ is the true value of the considered parameter. The true HR was used as the true value for the HR estimation and for the IDR estimation because the goal of the study was to evaluate the adequacy of the IDR as approximation of the HR. Moreover, in the case of non-constant hazard functions the IDR can be calculated by means of formula (5). However, there is no clear theoretical parameter available that is estimated by the empirical IDR.

The primary performance measure is given by the CP, which should be close to the nominal level of 95%. To identify data situations in which the IDR can be used as adequate approximation of the HR we used the criterion that the CP of the 95% CI should be at least 90%. A rule of thumb was developed depending on the relative average follow-up duration in the control group and the baseline risk, to decide whether or not the IDR can be used as a meaningful approximation of the HR.

## Results

### Simulation study

In the situations considered in the simulation study it is not problematic to use the IDR as approximation of the HR if the average follow-up durations in both groups are equal and the BLR is not larger than 25%. The minimum CP of the interval estimation of the IDR is 92,5% (CP for HR 93,4%) for the Weibull and 91,2% (CP for HR 93,1%) for the Gompertz distribution. There were no relevant differences between the IDR and HR estimations in bias or MSE (results not shown). This means that even in the case of non-constant hazard functions but a constant HR, the IDR - independent of the effect size and the sample size - can be used as approximation to the HR if the average follow-up durations in both groups are equal and the BLR is not larger than 25%.

The situation is different in the case of unequal average follow-up durations in the two groups, which is the more important case in practice. In this situation, there are shortfalls in the CP and in part large relative bias values for the IDR. The CP decreases remarkably under the nominal level of 95% with increasing difference in the average follow-up durations between the groups. The CP improves with decreasing sample size, due to the decreasing precision. Therefore, the sample size of *N* = 1000 is the relevant situation for the derivation of general rules.

**Fig. 1:**
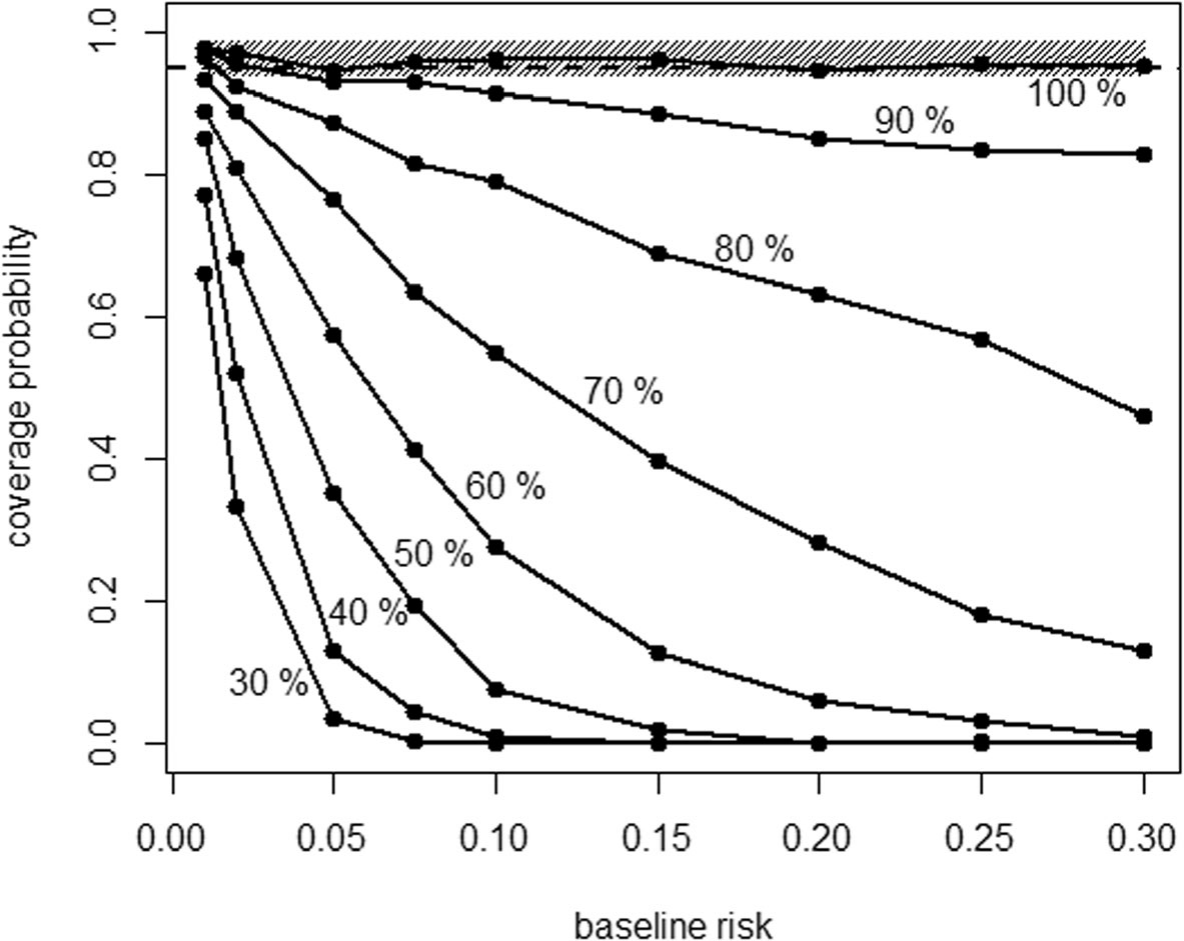
Coverage probability (CP) by baseline risk for the Gompertz distribution with shape parameter α = 1, sample size *N* = 1000, relative average follow-up duration in the control group from 30% to 100%, and a true hazard ratio (HR) of 0.4. The shaded area is the range of the CP for the HR over all these 72 scenarios; solid lines represent the CP for the incidence density ratio (IDR) for the different relative average follow-up duration in the control group; the horizontal dashed line marks the desired CP of 0.95

Figure 1 shows exemplarily the CP results for IDR and HR dependent on the BLR and the relative average follow-up duration in the control group, for the Gompertz distribution with shape parameter α = 1, sample size *N* = 1000, and a true HR of 0.4. We see that the CP for the IDR decreases remarkably under the nominal level of 95% with increasing difference in the average follow-up durations between the groups and with increasing BLR, whereas the CP for the HR lies within the desired area in all situations.

The results for the Gompertz distribution, with shape parameter α = 1, sample size *N* = 1000, and a relative average follow-up duration in the control group of 90%, are presented In Table 1 as an example. We can see in Table 1 that the CP of the 95% CIs of the IDR is larger than 90% if BLR is ≤ 10%, but is below 90% if BLR is ≥ 15%, which means that IDR is an adequate approximation of the HR in the corresponding data situation if BLR is ≤ 10%. However, even in these cases a strong, relative bias in the IDR occurs with absolute MPE values partially above 100% (overestimation for the Weibull and underestimation for the Gompertz distribution). This can be accepted in practice for the following reason. The MPE is given in the log scale. A relative bias of MPE = 100% means that a true HR = 0.9 is estimated by IDR = 0.81. Such a bias seems to be acceptable if the corresponding CI has a CP of at least 90%.

**Table 1:** Results for the Gompertz distribution

BLR	True HR	CP		MPE		MSE		SE	
		IDR	HR	IDR	HR	IDR	HR	IDR	HR
0.01	0.4	0.976	0.978	−22.678	−9.860	0.595	0.580	0.026	0.027
	0.7	0.964	0.978	−45.271	−5.351	0.612	0.634	0.023	0.024
	0.9	0.983	0.989	− 128.131	1.149	0.466	0.461	0.021	0.022
	1	0.977	0.976	NA	–	0.458	0.455	0.020	0.021
0.02	0.4	0.956	0.970	−7.247	7.156	0.369	0.404	0.018	0.019
	0.7	0.952	0.956	−34.036	1.679	0.280	0.285	0.015	0.016
	0.9	0.943	0.953	− 118.042	4.062	0.240	0.243	0.014	0.015
	1	0.956	0.973	NA	NA	0.209	0.214	0.014	0.014
0.05	0.4	0.930	0.948	−11.000	3.343	0.145	0.149	0.011	0.012
	0.7	0.928	0.964	−35.534	1.250	0.098	0.091	0.009	0.010
	0.9	0.936	0.966	− 133.290	−13.655	0.095	0.083	0.009	0.009
	1	0.929	0.946	NA	NA	0.087	0.077	0.009	0.009
0.075	0.4	0.931	0.970	−12.835	2.092	0.086	0.082	0.009	0.009
	0.7	0.921	0.958	− 37.182	−1.180	0.070	0.059	0.008	0.008
	0.9	0.914	0.954	− 125.979	−6.983	0.069	0.055	0.007	0.007
	1	0.916	0.945	–	–	0.065	0.053	0.007	0.007
0.1	0.4	0.914	0.943	−11.975	2.503	0.076	0.072	0.008	0.008
	0.7	0.907	0.941	−33.896	2.140	0.061	0.052	0.007	0.007
	0.9	0.927	0.968	−102.743	13.059	0.047	0.038	0.006	0.006
	1	0.902	0.959	NA	NA	0.053	0.038	0.006	0.006
0.15	0.4	**0.885**	0.942	−14.697	0.333	0.058	0.046	0.006	0.006
	0.7	**0.875**	0.943	−35.599	0.407	0.045	0.033	0.005	0.006
	0.9	**0.888**	0.953	− 115.852	0.054	0.039	0.027	0.005	0.005
	1	**0.884**	0.958	NA	NA	0.037	0.024	0.005	0.005
0.2	0.4	**0.851**	0.949	−15.946	−1.037	0.049	0.031	0.005	0.006
	0.7	**0.852**	0.945	−36.576	−1.049	0.037	0.023	0.005	0.005
	0.9	**0.869**	0.955	− 111.602	0.545	0.031	0.019	0.004	0.004
	1	**0.862**	0.951	NA	NA	0.031	0.019	0.004	0.004
0.25	0.4	**0.835**	0.957	−15.713	−0.142	0.043	0.025	0.005	0.005
	0.7	**0.830**	0.951	−36.719	−0.629	0.033	0.019	0.004	0.004
	0.9	**0.854**	0.950	−115.785	−5.196	0.028	0.015	0.004	0.004
	1	**0.872**	0.956	NA	NA	0.024	0.015	0.004	0.004
0.3	0.4	**0.829**	0.950	−16.209	0.014	0.038	0.019	0.004	0.004
	0.7	**0.818**	0.956	−36.302	− 0.295	0.029	0.014	0.004	0.004
	0.9	**0.862**	0.946	− 103.272	6.879	0.023	0.013	0.004	0.004
	1	**0.857**	0.948	NA	NA	0.021	0.013	0.003	0.004

Gompertz distribution with shape parameter α = 1, sample size *N* = 1000, and a relative average follow-up duration of 90% in the control group

If the true HR is 1 the MPE cannot be calculated

*BLR* baseline risk, *CP* coverage probability, *HR* hazard ratio, *IDR* incidence density ratio, *MPE* mean percent error, *MSE* mean square error, *SE* standard error

Numbers in boldface indicate a CP below 90%

Thresholds for BLR were derived for all other data situations. In total, 4 × 3 × 8 = 96 tables were produced for the 4 survival-time distributions, 3 sample sizes, and 8 relative average follow-up durations considered in the control group. The results are summarized in Table 2. Whether the IDR can be considered as adequate approximation of the HR depends not only on the BLR and the difference in the average follow-up durations between the groups but also, e.g., on the true survival-time distribution, which is unknown in practice. However, to derive general rules for the identification of situations in which the IDR can be used as approximation for the HR, the consideration of the BLR in dependence on the relative average follow-up duration in the control group seems to be sufficiently accurate. From Table 2, the following pragmatic rules can be derived:

- The IDR can be used in the case of equal follow-up durations in the two groups if BLR is ≤ 25%
- The IDR can be used in the case of a relative average follow-up duration in the control group between 90% and 100% if BLR is ≤ 10%
- The IDR can be used in the case of a relative average follow-up duration in the control group between 50% and 90% if BLR is ≤ 1%
- The IDR should not be used in the case of relative average follow-up durations < 50% in the control group

**Table 2:** Maximum BLR for which CP of at least 90% is reached for interval estimation of IDR as approximation of the HR

Relative average follow-up time of the control group	Maximum BLR			
	Weibull (decreasing hazard)	Gompertz (increasing hazard)		
		α = 0.5	α = 0.75	α = 1
30%	–	1%	–	–
40%	–	1%	–	–
50%	1%	2%	1%	–
60%	2%	2%	1%	1%
70%	7.5%	5%	2%	1%
80%	30%	10%	2%	2%
90%	30%	30%	20%	10%
100%	30%	30%	30%	25%

Total sample size *N* = 1000

*BLR* baseline risk, *CP* coverage probability, *HR* hazard ratio, *IDR* incidence density ratio

Other improved rules can be derived in certain situations if there is knowledge about the true survival-time distribution. However, this requires new simulations with the specific survival-time distribution. Without knowledge about the true survival-time distribution, the rule of thumb presented above can be used for practical applications when there is no access to the individual patient data.

### Example

For illustration we consider the IQWiG dossier assessment, in which the added benefit of enzalutamide in comparison with watchful waiting was investigated in men with metastatic prostate cancer that is not susceptible to hormone-blocking therapy, who have no or only mild symptoms, and in whom chemotherapy is not yet indicated [12]. According to the overall assessment, enzalutamide can prolong overall survival and delay the occurrence of disease complications. The extent of added benefit is dependent on age [12].

The benefit assessment was based upon an RCT, which was the approval study for enzalutamide in the indication described above. In this study, patients were randomized to either enzalutamide (intervention group) or placebo (control group), while the hormone-blocking therapy was continued in all patients. In each group, treatment was continued until either disease progression or safety concerns arose. Due to differential treatment discontinuation by treatment group, the median follow-up duration for safety endpoints was threefold longer in the intervention group (17,1 months) compared to the control group (5,4 months).

Here, we consider the endpoint hot flashes, which played a minor role in the overall conclusion of the benefit assessment. However, for the present study this endpoint is relevant, because interesting results are available for three different analyses. In the corresponding dossier submitted by the company, effect estimates with 95% CIs and *P* values were presented in the form of risk ratios (RRs) based upon naive proportions, as IDRs and as HRs. Additionally, Kaplan-Meier curves were presented. In each of the analyses only the first observed event of a patient was counted, i.e., there are no problems due to neglect of within-subject correlation.

The following results were presented in the dossier for the endpoint “at least one hot flash”. In the intervention group 174 (20.0%) among *n*_1_ = 871 patients experienced one or more events compared to 67 (7.9%) among *n*_0_ = 844 patients, which leads to an estimated RR = 2.52 with 95% CI 1.93, 3.28; *P* < 0.0001. However, as correctly argued by the company, this statistically significant effect could be induced simply by the threefold longer median follow-up duration in the control group. To account for the differential follow-up duration by treatment group, events per 100 patient years were presented (14.7 in the intervention group and 12.4 in the control group) leading to the not statistically significant result of IDR = 1,19 with 95% CI 0.87, 1.63; *P* = 0.28. However, according to our pragmatic rules, the IDR should not be used if the relative average follow-up duration in the control group is below 50%, which is the case here. Therefore, the validity of the IDR results is questionable in this example. Fortunately, the results of the Cox proportional hazards model were also presented. The result was statistically significant with an estimated HR = 2.29, 95% CI 1.73, 3.05; *P* < 0.0001. It should be noted that censoring is possibly not independent of outcome, leading to high risk of bias. Nevertheless, the results of the Cox proportional hazards model are interpretable and were accepted in the dossier assessment with the conclusion of a considerable harm of enzalutamide for the endpoint hot flashes [12].

This example shows that the use of IDR is invalid in the present case of differential follow-up duration by treatment group and non-constant hazard functions. From the Kaplan-Meier curves presented in the dossier it can be concluded that the hazard function of the endpoint hot flashes is decreasing. This situation can be illustrated as follows.

**Fig. 2:**
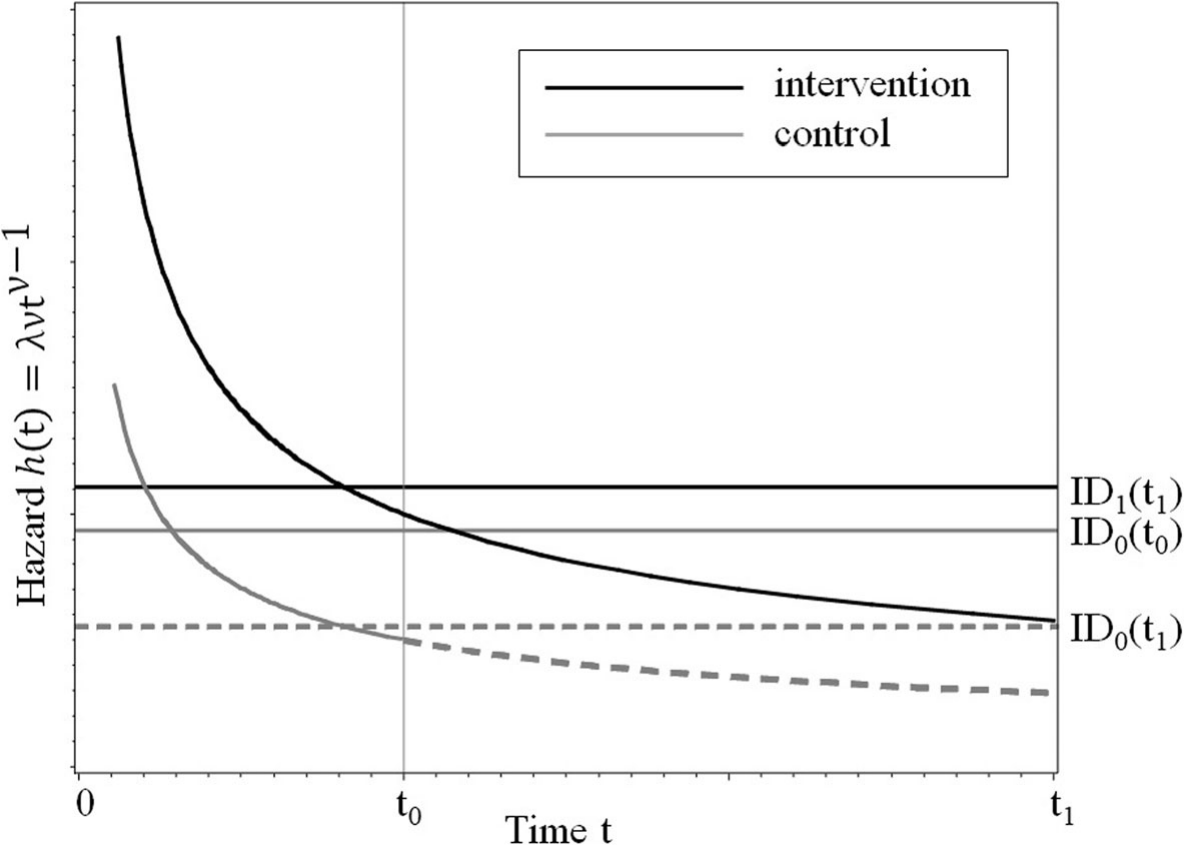
Effect of a shorter follow-up duration in the control group on the incidence density ratio (IDR). ID_1_(t_1_) is the estimated average hazard in the intervention group up to t_1_ (black solid line), ID_0_(t_0_) is the estimated average hazard in the control group up to t_0_ (gray solid line); ID_0_(t_1_) is the estimated average hazard in the control group up to t_1_ (gray dashed line), which is not observed; the use of ID_1_(t_1_) and ID_0_(t_0_) leads to a biased estimate of the hazard ratio (HR)

In Fig. 2 we consider the situation of decreasing hazard with true HR = 2, i.e., the hazard in the intervention group is larger compared to the control group. The relative average follow-up duration in the control group is only 33% compared to the intervention group. If the hazard is estimated simply by means of events per person year, it is implicitly assumed that the hazards are constant. In fact, however, the average hazard in each group is estimated by means of the ID for the available follow-up duration. As the follow-up duration in the control group is much shorter, the right part of the true hazard function is not observed, which leads to a strong bias of the ID as estimate of the average hazard in the control group. Therefore, the IDR is also biased as an estimate of the HR. In this example with decreasing hazards and a large difference in the follow-up durations between the treatment groups, the harmful effect of enzalutamide on the endpoint hot flashes in comparison with watchful waiting could not be detected by means of the IDR. Therefore, the IDR is invalid here and should not be used to describe the effect of the intervention.

## Discussion

The IDR represents a valid estimator of the HR if the true hazard function is constant. However, for non-constant hazard functions we found that in the simulated data situations with decreasing and increasing hazard functions, the IDR is only an adequate approximation of the HR if the average follow-up durations in the groups are equal and the baseline risk is not larger than 25%. In the case of differential follow up by treatment group, the validity of the IDR depends on the true survival-time distribution, the difference between the average follow-up durations, the baseline risk, and the sample size. As a rule of thumb, the IDR can be used as approximation of the HR if the relative average follow-up duration in the control group is between 90% and 100% and BLR is ≤ 10, and in the situation where the average follow-up duration in the control group is between 50% and 90% and BLR is ≤ 1%. The IDR should not be used for relative average follow-up durations in the control group below 50%, because in general the IDR represents no valid approximation of the HR and the meaning of the IDR is unclear. The usefulness of this rule of thumb was illustrated by means of a real data example.

The results and the conclusions of our simulation study are limited in the first instance to the data situations considered. We considered a wide range of effect sizes (HR 0.4–2.5), three total sample sizes (*N* = 200, 500, 1000) with balanced design, and four survival-time distributions with deceasing (Weibull distribution) and increasing hazard functions (Gompertz distribution). For the baseline risk, we considered almost the complete range (0.01–0.99) in the simulations. We derived practical rules to decide in which data situations the IDR can be used as approximation of the HR. These rules should also be approximately valid for other data situations. If detailed knowledge of the underlying survival-time distribution is available, more simulations can be performed to find improved rules for the specific data situation.

We have not investigated the amount of bias associated with different patterns of dependent censoring. In this context, the framework of estimands offers additional possibilities to deal with competing events, leading to censoring mechanisms that are not independent of the considered time-to-event endpoint [13]. We have also not considered the data situations with recurrent events. Extensions of the Cox proportional hazards model, such as the Andersen-Gill, the Prentice-Williams-Peterson, the Wei-Lin-Weissfeld, and frailty models [14, 15] have been developed for analysis of recurrent event data. The application of methods for analysis of recurrent event data to analysis of adverse events in RCTs is discussed by Hengelbrock et al. [16]. Further research is required for the investigation of the impact of dependent censoring and multiple events on the validity of the IDR.

## Conclusions

In summary, in the case of large differences in the average follow-up durations between groups, the IDR represents no valid approximation of the HR if the true hazard functions are not constant. As constant hazard functions are rarely justified in practice, adequate survival-time methods accounting for different follow-up times should be used to analyze adverse events rather than the simple IDR, including methods for competing risks [17]. However, the proposed rule of thumb allows the application of IDR as approximation of the HR in specific data situations, when it is not possible to estimate the HR by means of adequate survival-time methods because the required individual patient data are not available.

## Data Availability

All results from the simulated data are available from the authors on reasonable request. The data presented in the examples are available online [6, 12].

## Abbreviations

BLR: Baseline risk
CI: Confidence interval
CP: Coverage probability
EAIR: Exposure-adjusted incidence rate
HR: Hazard ratio
ID: Incidence density
IDR: Incidence density ratio
IQWiG: Institut für Qualität und Wirtschaftlichkeit im Gesundheitswesen
MPE: Mean percent error
MSE: Mean square error
RCT: Randomized controlled trial
RR: Risk ratio
SE: Standard error
